# Is a Problem-Solving Intervention with Workplace Involvement for Employees on Sickness Absence Due to Common Mental Disorders More Effective, than Care as Usual, in Reducing Sickness Absence Days? Results of a Cluster-Randomised Controlled Trial in Primary Health Care

**DOI:** 10.1007/s10926-024-10229-4

**Published:** 2024-08-07

**Authors:** Ida Karlsson, Anna Frantz, Iben Axén, Gunnar Bergström, Ute Bültmann, Anna Finnes, Kristina Holmgren, Lydia Kwak, Elisabeth Björk Brämberg

**Affiliations:** 1 Institute of Environmental Medicine, Unit of Intervention and Implementation Research for Worker Health, Karolinska Institutet, Stockholm, Sweden; 2 Department of Occupational Health, Psychology and Sports Sciences, Faculty of Health and Occupational Studies, University of Gävle, Gävle, Sweden; 3 Department of Health Sciences, Community and Occupational Medicine, University Medical Center Groningen, University of Groningen, Groningen, The Netherlands; 4 Division of Psychology, Department of Clinical Neuroscience, Karolinska Institutet, Stockholm, Sweden; 5 Academic Primary Health Care Centre, Region Stockholm, Stockholm, Sweden; 6 Department of Health and Rehabilitation, Institute of Neuroscience and Physiology, Sahlgrenska Academy, University of Gothenburg, Gothenburg, Sweden; 7 School of Public Health and Community Medicine, Institute of Medicine, University of Gothenburg, Gothenburg, Sweden

**Keywords:** Work-directed intervention, Depression, Anxiety disorder, Adjustment disorder, Problem-solving intervention, Primary health care

## Abstract

**Purpose:**

The aim was to evaluate the effectiveness of a problem-solving intervention with workplace involvement (PSI-WPI) added to care as usual (CAU) in reducing sickness absence days among employees with common mental disorders compared to CAU alone in Swedish primary health care on a monthly basis over 18-months follow-up.

**Methods:**

We conducted a cluster-randomised controlled trial including 197 employees blinded to allocation (85 PSI-WPI and 112 CAU). As sickness absence data was skewed and over-dispersed, generalised estimating equations was used to enable a comparison between the intervention and control group for each month of the follow-up period.

**Results:**

The median number of sickness absence days over the 18-month follow-up was 78 days, inter-quartile range (IQR) 18–196 for employees receiving PSI-WPI and 64 days, IQR 18–161 for employees receiving CAU. The time x group generalised estimating equations analysis showed no statistically significant difference in sickness absence days per month.

**Conclusion:**

The addition of a PSI-WPI to CAU was not more effective in reducing sickness absence days. This may be explained by the primary health care context, lack of specialisation in occupational health and the Swedish social insurance system with specific time limits.

Trial registration.

The trial was registered at ClinicalTrials.gov, identifier: NCT03346395 on January 12th, 2018.

## Introduction

Common mental disorders (CMDs), i.e. mild to moderate depression, stress-related disorders and adjustment disorders affect approximately one in six people of working age in the European Union each year [1]. CMDs increase the risk of sickness absence (SA) and SA due to a CMD result in longer SA episodes compared with non-CMD diagnoses [2]. In Sweden, the risk of SA due to CMDs is 10–15% higher in the age group 25–39 compared to older age groups, deviating from the general trend of increasing SA risk with increasing age for non-CMD diagnoses [3]. In addition, CMDs cause individual suffering and economic losses, productivity loss for employers and increased societal costs due to health care expenses and social insurance benefits costs [4]. Finding effective interventions to reduce SA among employees with CMDs is therefore needed for individuals, employers and society at large.

Clinical interventions for CMDs usually include psychological treatment such as cognitive behavioural therapy and/or pharmacological interventions [5]. These interventions often result in decreased symptoms but have little or no effect on SA or return to work (RTW) [6–9]. Combining psychological interventions with workplace involvement have shown promising results in reducing SA among workers with depressive disorders [10] and CMDs [11]. One such intervention is a problem-solving intervention with work-focus aimed at increasing the employee’s problem-solving skills to manage the work situation and RTW process [12]. When provided in occupational health services (OHSs) the work-focused problem-solving intervention has shown promising effects in reducing total SA days [6, 12, 13], preventing recurrent SA episodes for employees with CMDs [14] and shortening the time until partial RTW for employees with CMDs over 12 months follow-up [15]. These results have been reported from OHS settings in countries (mainly the Netherlands) that have different social insurance systems than Sweden and effectiveness evaluations beyond 12 months are lacking [10, 13–15].

The Swedish health care system is commissioned to support employees on SA with RTW and to facilitate collaboration between the stakeholders involved in the RTW process, by offering coordinator services, for example through rehabilitation coordinators (RCs) [16]. In Sweden, interventions supporting employees on SA with RTW have commonly been provided by the OHS. However, only around 60% of employees in Sweden, mostly employed in medium to large enterprises, have access to the OHS [17]. Compared to the Swedish OHS, all citizens have access to primary health care (PHC) which is also the first-line psychiatry for persons with CMDs [18]. People with different physical and mental symptoms related to work often consult the PHC at an early stage and generally before considering SA [19]. However, the PHC does not have a history of providing work-focused interventions or involving their patients’ workplaces in the RTW process. With regards to the OHS, the first contact with the OHS is initiated by the workplace (e.g. the first-line manager), in comparison to the PHC in which the contact is initiated by the employee, and involvement of the workplace is only initiated if the patient consent. Recently, the randomised controlled trial (RCT) conducted by Keus van de Poll et al. [12] evaluated the effect of a problem-solving intervention with workplace involvement provided by the Swedish OHSs on SA days among employees with CMDs or stress-related symptoms. The RCT showed that the intervention group had 15 days less SA over a 12-month follow-up period, compared to a control group receiving care as usual (CAU) [12]. Based on the study by Keus van de Poll et al. [12], a cluster RCT was designed to evaluate the effect of a problem-solving intervention with workplace involvement (PSI-WPI) in Swedish PHC [20]. Previous studies have recommended interventions combining a clinical intervention with a work-directed intervention [10]. Further, there is an initiative from the Swedish government to strengthen the focus on workplace involvement in PHC [16]. It is therefore of interest to evaluate if the effective problem-solving intervention provided by the Swedish OHS can be translated to the Swedish PHC.

The current RCT applied a clustered design to include employees from several PHC centres with different care-need-indexes [21]. The aim of the study was to evaluate the effectiveness of a PSI-WPI added to CAU in reducing SA days among employees with CMDs compared to CAU alone in Swedish PHC on a monthly basis over 18 months follow-up.

## Method

### Trial Design and Setting

The study was designed as a two-armed cluster RCT carried out in PHC in the Västra Götaland region of Sweden over 18 months follow-up. The study design and setting have been described elsewhere [20]. The trial was registered at ClinicalTrials.gov on January 12th, 2018, identifier: NCT03346395. The reporting of the study follows the CONSORT guidelines extended to cluster RCTs [22].

***Ethical Approval and Consent to Participate.***

The Swedish Ethical Review Authority approved the study on June 21st, 2017, reference number 496–17. All Participants received oral and written information about the study and signed a written informed consent upon enrolment. The study was conducted in accordance with the World Medical Association, Declaration of Helsinki [23].

### Recruitment, Randomisation and Blinding

For the eligibility and recruitment of the RCs see Björk Brämberg et al. [20]. Randomisation was conducted at RC level (one RC was defined as one cluster) using a random number allocator. RCs were not blinded to allocation but instructed not to reveal information about the content of PSI-WPI to the participants. Eighty RCs received information about the study and the final sample consisted of 19 RCs (9 PSI-WPI and 10 CAU), covering 24 PHC centres (three RCs covered two PHC centres and one RC covered three PHC centres). Participants were recruited through screening of medical records by a PHC assistant in agreement with the PHC management. Eligible participants received written information about the study and an invitation to participate by post. The information given was the same for both groups, to ensure blinding. If the participant consented to participation, a written informed consent was signed and returned to the PHC assistant in a prepaid envelope. Thereafter, the consent was passed to the principal investigator who contacted the RC at the participants PHC centre. The participant was then contacted by the RC and consequently followed the randomisation of the RC.

### Inclusion and Exclusion Criteria

Participants were eligible if they were currently employed, aged between 18 and 59 years, on SA for 2 to 12 weeks due to a diagnosis of mild to moderate depression, anxiety, or adjustment disorder as the primary cause of SA and diagnosed by a physician at one of the participating PHC centres. In addition they had to accept employer involvement in the RTW process and understand written and spoken Swedish. Exclusion criteria were a diagnosis of severe depression, acute stress reaction, post-traumatic stress, or any other severe mental disorder such as psychotic or bipolar disorder or referral to a psychiatrist, pregnancy, somatic complaints, or other disorders that could affect ability to work.

### The Problem-Solving Intervention with Workplace Involvement (PSI-WPI)

The PSI-WPI is a structured procedure for active problem-solving that integrate the participants own ideas in the treatment [24], build on a participatory approach and involves the participants workplace manager in the RTW process [25]. The intervention was delivered by RCs to the participant (hereafter referred to as employee) in a minimum of two to approximately five sessions. The intervention usually started within one week after enrolment. No specific timeframe was set for the intervention. RCs received a 2-day training, a manual and worksheets prior to the intervention. For a detailed description of the training, see Björk Brämberg et al. [20].

The PSI-WPI comprised a structured five-step problem-solving process with workplace involvement in addition to CAU. The first step involved a meeting between the RC and the employee, at which an inventory was made of the employee’s situation, e.g. the reason for SA, as well as private- and work-related problems impacting RTW, from the perspective of the employee. After the inventory, the RC contacted the manager by phone to explain the PSI-WPI method and to make an inventory of the manager’s view of the employee’s problems at work and the reason for SA. In the second step, the RC and employee brainstormed about solutions to the previously discussed problems and about topics to be addressed during the upcoming meeting with the manager. In the third step, the RC and employee formulated an action plan built on the identified problems, in which proposed solutions and the employees’ need for support to be able to implement the proposed solutions were described. The fourth step comprised a three-part meeting between the RC, the employee and the manager, with the aim to agree on the action plan and to discuss potential additional solutions and the need of work accommodation. In the fifth step, the employee implemented the action plan, and the RC and employee evaluated the progress. The manager could be involved in the evaluation but did not have to be. If necessary, the steps could be repeated [20].

### Care as Usual

In Sweden, CAU concerning depression and anxiety disorders should follow recommendations from the National Board of Health and Welfare usually consisting of cognitive behaviour therapy or antidepressant medication [5]. There are so far no such guidelines for the treatment of adjustment disorders. However, access to psychological treatment in Swedish PHC generally does not correspond to demand. The content of CAU can therefore not be clearly described but reflects the typical treatment range in Swedish PHC. Further, CAU could include strategies for RTW, e.g. contact with the employee’s workplace. All employees received CAU, irrespective of randomisation.

### Outcome Measures

The primary outcome was the number of registered net SA days, i.e. full-time SA days over the 18-month follow-up period. The mean net SA days were calculated monthly to compare PSI-WPI and CAU. The registry data was collected from the MicroData for the Analysis of Social insurance (MiDAS) register provided by the Swedish Social Insurance Agency [26]. The register contains information on SA days that exceed the 14th SA day, as well as part-time or full-time sickness compensation (25%, 50%, 75% or 100% of regular working hours). Data for the primary outcome was collected 18-months after, and 24-months prior to inclusion.

### Information Collected at Baseline

Diagnosis upon enrolment was available in the MiDAS register. All included employees received a baseline questionnaire regarding age, gender, education, family situation, work sector, general health and psychological symptoms. The Hospital Anxiety and Depression scale (HAD) was used to assess symptoms of anxiety and depression [27], the stress-related Exhaustion Disorder (s-ED) instrument was used to assess stress-induced exhaustion [28], the Karolinska sleep questionnaire was used to assess sleep quality [29] and the Euro-QoL health state questionnaire (EQ-5D) was used to assess self-rated health [30]. Intention to RTW was measured by the question: “Do you intend to return to work even if you continue to have symptoms of stress, exhaustion, depression, or anxiety?” [31].

### Sample Size

The sample size calculation was based on the primary outcome, i.e. the number of net SA days over the 18-month follow-up period. The sample size was estimated to have 80% power to show a difference of at least 20% registered net SA days over 18 months from baseline between PSI-WPI and CAU [32, 33]. It was calculated that 10 RC clusters were needed with approximately 11 employees per cluster in the PSI-WPI and CAU groups, respectively, resulting in a total of 220 participating employees, approximately 110 in each group. An intra-cluster correlation was set to 0.01 with an *α* level of 0.05. No interim analyses or stopping guidelines were used.

### Data Analysis

The analysis was planned to be conducted on cluster level, but this was not possible because one cluster (RC level) only had one employee. Instead, the analysis was conducted on employee level that accounted for within-subject correlation. The analysis was conducted as an intention to treat analysis. For the descriptive statistics IBM SPSS statistics, version 28.01.1 was used [34]. For the analyses and graphs, RStudio 2023 (R version 4.2.3, (2023-03-15) was used [35]. To prepare the dataset with registered SA days for analysis, part-time SA days were transformed into full-day SA days, i.e. net SA days. The difference in total number of net SA days over the 18-month follow-up between the groups was not analysed due to a skewed distribution and over-dispersion. Through generalised estimating equations (GEE) the estimated mean and mean difference in net SA days per month and group was visualised and compared. An independent correlation structure was chosen based on the quasi-likelihood under the independence model criterion (QIC). Further, robust standard errors and an alpha level of 95% were used to examine the monthly difference in mean SA days between the groups. In the analysis, SA days were rounded to integers and treated as count data, the dependent variable was mean net SA days per month and the independent variable was group (PSI-WPI or CAU). Based on previous studies [36–38], two additional variables were created and adjusted for; if the follow-up period covered the COVID-19 pandemic, i.e. from March 1st, 2020 to September 1st_,_ 2021 with response format yes/no [36] and SA for ≥ 60 days 24 months prior to the index date (e.g. date of inclusion), which has been associated with an increased risk of future SA, with response format yes/no [37, 38]. Baseline was set to 30 days before the date of inclusion to have an independent baseline measure of SA days before enrolling in the RCT. Treatment group x time were estimated for months from 1 to 18. The GEE model was used to compute the mean difference in SA days per month and group. The log odds ratio of the difference was exponentiated to a ratio and reported with the estimated confidence interval. The final GEE analysis included the dependent and independent variable and an interaction term for group x time. Adjusting for sex, follow-up during the COVID-19 pandemic and ≥ 60 days on SA before baseline did not have a significant effect on the outcome. These variables were therefore removed from the final analysis to decrease the number of parameters and increase power.

## Results

**Fig. 1:**
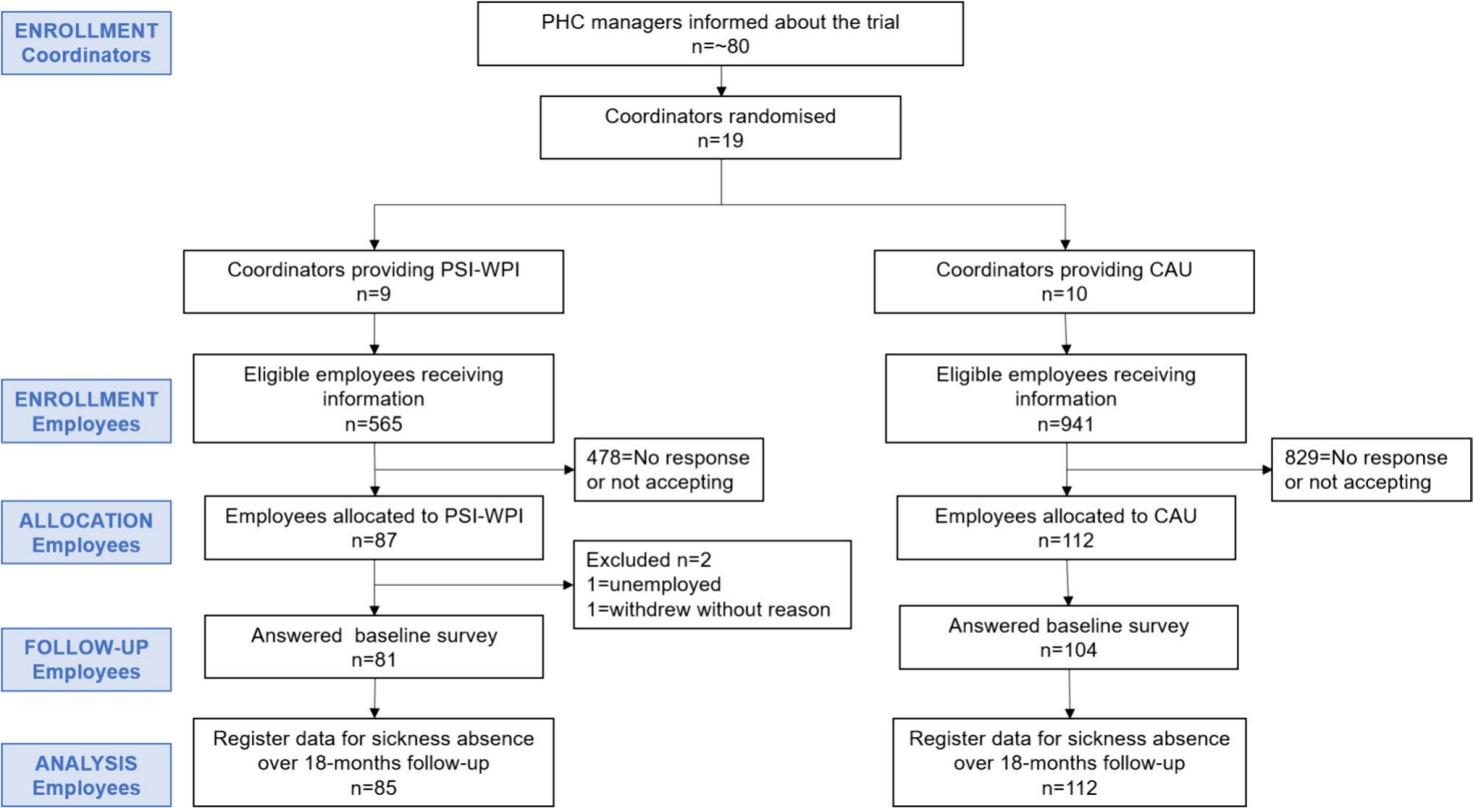
Flow-chart of employee recruitment. *PSI-WPI* Problem-solving intervention with workplace involvement, *CAU* care as usual

During the recruitment period (February 2018 to February 2020), 1506 eligible employees received written information about the study, out of which 199 consented to participate. Two employees were excluded, one employee due to unemployment, and the other employee withdrew without giving a reason. The final sample included 197 employees, 85 in the PSI-WPI and 112 in the CAU group (Fig. 1). The reason for employees not accepting participation is not known but among the eligible employees not consenting to participate, 74% were females and the mean age was 40-years for PSI-WPI while the respective proportion for CAU was 74% females with a mean age of 43-years. The 9 RCs providing PSI-WPI had a mean age of 57 years (range 39–68), all were female, 5 had worked as a RC for > 3 years and their professions were occupational therapist, physiotherapist, registered nurse, or was unknown. The 10 RCs providing CAU had a mean age of 52 years (range 33–64), 8 were female, 7 had worked as a RC for > 3 years and their professions were occupational therapist, physiotherapist, registered nurse, counsellor or was unknown. The 9 PSI-WPI clusters had a median number of employees of 9 (range 1–19) and the 10 CAU clusters had a median number of 11 employees (range 5–28).

### Attrition

The data collected on SA days for the employees was derived from the MiDAS register and thus no data on the SA outcome was missing.

### Baseline Data

The median age of employees was 42-years and 85% were females. The distribution of baseline characteristics was similar for most employees receiving PSI-WPI and CAU. Most employees had permanent employment (90% PSI-WPI vs. 96% CAU), and a similar proportion reported symptoms of anxiety (73% vs. 70%) and depression (64% vs. 64%). A few differences were found between the groups, for example a higher proportion of employees in the PSI-WPI group were diagnosed with adjustment disorder compared to CAU (60% vs. 46%) and a lower proportion was diagnosed with mild to moderate depression (14% vs. 32%). Further, a higher proportion of employees in the PSI-WPI group had a managerial position (20% vs. 8%) (Table 1).

**Table 1:** Employee characteristics per treatment group, at baseline

Characteristic	PSI-WPI (*n* = 85)	CAU (*n* = 112)
Age, mean (SD)	42 (11)	42 (10)
Gender, *n* (%)	85	112
Female	72 (85)	95 (85)
Sickness absence diagnosis at baseline, *n* (%)	85	112
Mild to moderate depression	12 (14)	36 (32)
Anxiety disorder	22 (26)	24 (22)
Adjustment disorder	51 (60)	52 (46)
Children < 16 living at home, *n* (%)	79	103
Yes	44 (66)	59 (57)
Immigrant status, *n* (%)	79	103
Yes	4 (6)	9 (9)
Education, *n* (%)	80	102
Primary education	3 (4)	4 (4)
Secondary education	42 (42)	52 (51)
University/Higher education	35 (44)	46 (45)
Work sector, *n* (%)	79	103
Public	40 (50)	52 (51)
Private	32 (41)	33 (32)
State	5 (6)	10 (10)
Other	2 (3)	8 (7)
Employment terms, *n* (%)	79	103
Permanent employment	71 (90)	99 (96)
Temporary employment	8 (10)	4 (4)
Years at workplace, *n* (%)	79	103
Less than 1 year	17 (22)	13 (13)
1–2 years	11 (14)	30 (29)
3–5 years	19 (24)	24 (23)
> 5 years	32 (40)	36 (35)
Managerial position, *n* (%)	79	103
Yes	16 (20)	8 (8)
Intention to return to work^a^, *n* (%)	61	84
1. Not at all	9 (15)	14 (17)
2	9 (15)	14 (17)
3	20 (33)	22 (26)
4	7 (11)	12 (14)
5. Absolutely	16 (26)	22 (26)
Self-reported depression^b^, *n* (%)	79	102
No depression (0–7 points)	25 (32)	29 (28)
Mild depression (8–10 points)	22 (28)	32 (31)
Depression (> 10 points)	32 (40)	41 (40)
Self-reported anxiety^b^, *n* (%)	79	102
No anxiety (0–7 points)	14 (18)	23 (23)
Mild to moderate anxiety (8–10 points)	17 (21)	24 (23)
Anxiety (> 10 points)	48 (61)	55 (54)
Self-reported exhaustion^c^, *n* (%)	79	100
No exhaustion	11 (14)	12 (12)
Light/moderate exhaustion	11 (14)	16 (16)
Pronounced exhaustion	57 (72)	72 (72)
Self-rated health^d^, mean (SD)	61 43 (20)	82 48 (20)
Sleep quality^e^, mean (SD)	76 3.5 (1.1)	101 3.3 (1.4)

^a^Measured by the question: “Do you intend to return to work even if you continue to have symptoms of stress, exhaustion, depression, or anxiety?” ^b^Measured with HAD depression and anxiety scale. ^c^Measuered with the self-report instrument s-ED. ^d^Measured with EQ-5D visual analog scale which records the self-rated health status on a scale of 0–100. ^e^Measured with the Karolinska sleep questionnaire

### Intervention and Co-Interventions

A higher proportion of employees receiving PSI-WPI had contact with a RC (85% versus 43% in CAU) and participated in a three-part meeting (41% versus 10% in CAU). No differences were found between employees in PSI-WPI and CAU for co-interventions or contact with a psychologist or counsellor (Table 2).

**Table 2:** Intervention components

Variable	PSI-WPI (*n* = 85)	CAU (*n* = 112)
Contact with RC, *n* (%)	72 (85)	48 (43)
Three-part meeting, *n* (%)	35 (41)	11 (10)
^a^Co-intervention, *n* (%)	36 (42)	35 (31)
Contact with psychologist/counsellor, *n* (%)	62 (73)	83 (74)

^a^Co-interventions usually consisted of stress management, medical yoga and/or participating in a rehabilitation programme with a physiotherapist or occupational therapist

### Sickness Absence Days During Follow-Up

The primary outcome net SA days during follow-up was analysed for 85 employees in PSI-WPI and 112 employees in CAU. The median number of SA days over the 18-month follow-up was 78 days (IQR 18–196) for PSI-WPI and 64 days (IQR 18–161) for CAU. The GEE analysis did not show any significant difference between the groups over the 18-month follow-up, *p* = 0.384.

### Sickness Absence Days Development During Follow-Up

**Fig. 2:**
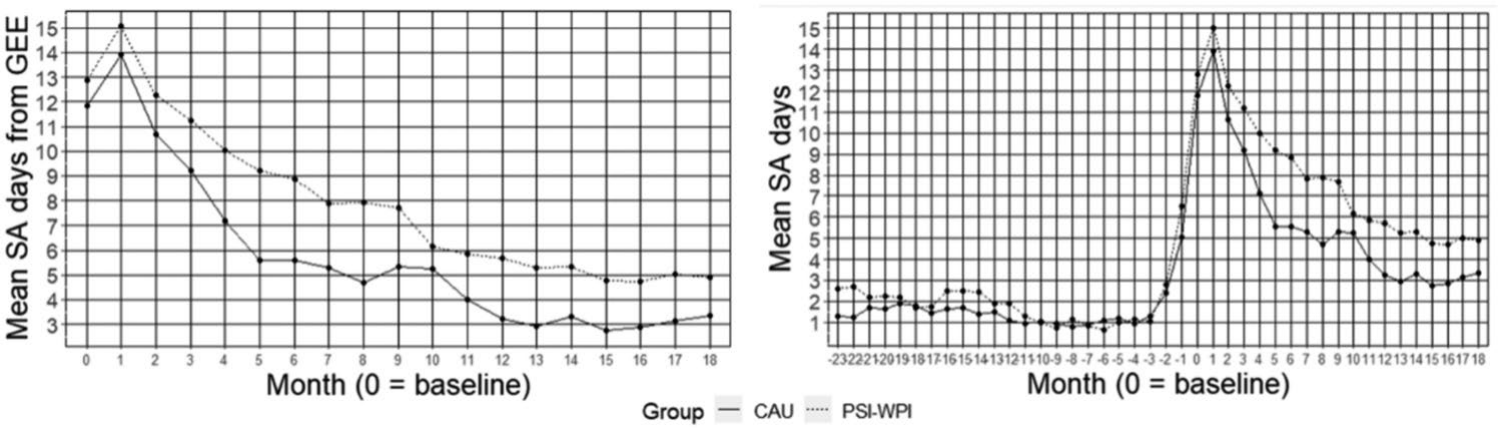
On the left, mean net sickness absence days per month estimated from GEE analysis. On the right, empirical mean net sickness absence days per month before and after baseline

Figure 2 (on the left) shows mean SA days per month estimated by GEE analysis. The time x group showed no difference in SA days over time, except for month five, *p* = 0.003. The estimated mean difference in SA days varied between 0.9 and 3.6 additional SA days per month for employees in PSI-WPI compared to CAU (Table 3). The differences were most prominent in month five (Ratio 1.64, 95% CI 1.11–2.43), month six (Ratio 1.59, 95% CI 1.07–2.36) and month eight (Ratio 1.68, 95% CI 1.06–2.67), all in favour of CAU. Figure 2 (on the right) shows empirical mean net SA days per month and group for 24-months before baseline.

**Table 3:** Generalised estimating equations analysis. Mean sickness absence days per month and group, estimated mean difference and an estimated ratio with 95% confidence intervals

Month	BL	1	2	3	4	5	6	7	8	9	10	11	12	13	14	15	16	17	18
Mean days (CAU)	11.8	13.9	10.7	9.2	7.2	5.6	5.6	5.3	4.7	5.3	5.2	4.0	3.2	2.9	3.3	2.7	2.9	3.1	3.4
Mean days (PSI-WPI)	12.9	15.1	12.3	11.2	10.0	9.2	8.9	7.9	7.9	7.7	6.2	5.9	5.7	5.3	5.3	4.8	4.7	5.0	4.9
^a^Mean difference	1.0	1.2	1.6	2.0	2.8	3.6	3.3	2.5	3.2	2.4	0.9	1.9	2.5	2.4	2.0	2.0	1.9	1.9	1.6
^b^Ratio	1.09	1.08	1.15	1.22	1.40	1.64^*^	1.59	1.48	1.68	1.45	1.18	1.46	1.76	1.81	1.60	1.74	1.65	1.60	1.46
95% CI lower	0.90	0.90	0.89	0.91	0.99	1.11	1.07	0.96	1.06	0.93	0.72	0.84	0.95	0.95	0.87	0.88	0.84	0.83	0.77
95% CI upper	1.31	1.31	1.49	1.63	1.96	2.43	2.36	2.27	2.67	2.27	1.93	2.55	3.27	3.45	2.96	3.46	3.26	3.08	2.79

^a^Mean difference; mean days, PSI-WPI–CAU. ^b^Ratio; exponentiation of the GEE log odds ratio with CAU as reference group

## Discussion

The aim of the study was to evaluate the effectiveness of a PSI-WPI added to CAU in reducing SA days among employees with CMDs compared to CAU alone in Swedish PHC on a monthly basis over 18-month follow-up. The results showed that PSI-WPI was not more effective in reducing SA days compared to CAU. Comparing the PSI-WPI and CAU group, did not show any significant effects.

The current trial builds on a previous Swedish RCT by Keus van de Poll et al. [12] and systematic reviews evaluating problem-solving interventions [10, 13, 15]. The earlier RCTs have mostly been conducted in OHS settings and have shown positive effects with regard to reducing SA by up to 25 days during the first year [10, 12]. Although the Swedish PHC setting play an important role in the RTW process for employees on SA, our RCT did not find an effect for the addition of PSI-WPI on top of CAU.

To date, the evaluated problem-solving interventions have mainly been provided by occupational health specialists such as RTW coordinators from OHS [39], occupational physicians [14], or OHS consultants [12]. Thus, the PSI-WPI partly introduced a ‘new way of working’ in PHC among RCs with limited training in work-focused interventions. The limited training and the PHC tradition of providing treatment (i.e. symptom reduction) might have resulted in a lack of contrast between the PSI-WPI and CAU, especially if the different steps of the intervention were not completed. In our qualitative study of PSI-WPI, the RCs experienced the intervention as supportive although more time-consuming than CAU [40]. Also, the three-part meeting involving the employee and his/her first-line manager was essential to enable a dialogue, to discuss the way forward and to identify potential disagreements between the employee and manager [40]. Although a higher proportion of employees in the PSI-WPI group had contact with a RC (85% vs 43%) and had a three-part meeting (41% vs 10%), these contacts and meetings may not be enough to make a difference in SA days between the intervention and control groups. A process evaluation is currently being conducted and will provide insight into adherence to the study protocol. This will allow a comparison of dose delivered and dose received (e.g. number of meetings) and the potential association with SA days.

Further, the study population of our RCT in the PHC setting differed from similar RCTs in the OHS setting [12, 14, 39]. The employees in the current RCT were on SA for 2 to 12 weeks at inclusion, while other RCTs included individuals that had or were soon expected to RTW [14], seeking support from the OHS, whether sick listed or not [12], or that were on SA for two to eight weeks [39]. Overall, in the previous RCTs the study populations were closer to RTW.

In view of the different populations and the RTW outcome, it could be that the timing of when to provide the PSI-WPI needs to be considered. In line with Dewa et al., more knowledge is needed to determine if there is an optimal time in the SA process for when an employee is susceptible to learn and gain from the problem-solving skills [41]. Next to the timing of the intervention, it may be that PSI-WPI would be more beneficial for certain diagnoses. Additional sub-group analyses of PSI-WPI may provide important information, although this would require a larger sample [42, 43]. The mean SA days per month decreased for employees in both groups. Up until month five, no significant differences between the PSI-WPI and CAU groups were observed. This may be explained by the Swedish Social Insurance System and its “rehabilitation chain”, in which the individual’s work ability is assessed for sickness benefit entitlement at specific timepoints [44]. In the rehabilitation chain the individual’s work ability is assessed in relation to the work role during the first 90 days of SA, in relation to any work role at the workplace after day 90, and to any work role available in the labour market, after day 180. Hence, the Swedish social security regulations may have had an impact on the decrease in SA days up until month five, which was visible in both groups, since it becomes more difficult to receive a SA certificate after six months [45]. Another potential explanation for the differences in SA length could also be the National Board of Health and Welfare guideline about the recommended SA length for each diagnosis [45]. The slightly higher proportion of employees diagnosed with adjustment disorder in PSI-WPI (60%) compared to CAU (46%) contributed to the skewed distribution in SA days between the groups. The guideline [45] prescribes the longest SA recommendation to exhaustion disorder (a sub-diagnosis to adjustment disorder), with up to 6 months (or up to 12 months if cognitive problems remain).

### Strengths and limitations

The study was conducted in a PHC setting under ‘real world’ conditions following the recommendations for RCTs, which is the gold standard for evaluating effectiveness. The strengths of the study are that the primary outcome was measured with registry data and thus data was objective and without loss to follow-up. In addition, the recruitment process of the employees was conducted by an independent PHC assistant, blinded to allocation. The study also has some limitations. Out of 1506 eligible employees, 199 (13%) consented to participate in the trial. In the information to eligible employees, all had to accept the involvement of their manager. It cannot be ruled out that employees involved in conflicts with their manager or those with a high symptom severity chose not to participate. Hence, this may affect the representativeness of the sample and in turn the generalisability of the results. Additionally, our sample consisted of mostly highly educated females. The planned sample size of 220 employees was not reached increasing the risk of type II error. Furthermore, the current study was a cluster RCT, but there was an imbalance in size between clusters, with the smallest containing only one employee. This meant that the analysis could not account for the cluster variable which may have resulted in an increased risk of type I error [46]. We cannot fully estimate the trade-off between these two risks of error in this study.

## Concluding Remarks

Our results suggests that the addition of a PSI-WPI to the CAU offered by PHC in Sweden to employees on SA due to CMDs, was not effective in reducing SA days. The translation of the PSI-WPI from the OHS setting to the PHC setting needs to be reconsidered. Factors such as the provider of the intervention, the included study population and the role of the Swedish social insurance system should be considered when planning PSI-WPI in the PHC setting.

## Data Availability

The data is not publicly available due to containing information that could compromise the privacy of the study participants. Reasonable inquiries about access may be sent to Karolinska Institutet, Institute of Environmental Medicine, Unit of Intervention and Implementation Research for Worker Health, Box 210, 171 77 Stockholm or by contacting the Research and Data Office at Karolinska Institutet: rdo@ki.se. The Swedish Ethical Review Authority will then be contacted for permission.

## Declarations

### Protocol

Björk Brämberg E, et al. Increasing return-to-work among people on sick leave due to common mental disorders: design of a cluster-randomized controlled trial of a problem-solving intervention versus care-as-usual conducted in the Swedish primary health care system (PROSA). BMC Public Health. 2018;18 (1):889 [20].
